# Medial pivot total knee arthroplasty for valgus knees provides equivalent medial stability compared to that for varus knees: In vivo kinematic study

**DOI:** 10.1002/jeo2.70013

**Published:** 2024-12-18

**Authors:** Tomofumi Kage, Kenichi Kono, Tetsuya Tomita, Takaharu Yamazaki, Shuji Taketomi, Ryota Yamagami, Kohei Kawaguchi, Ryo Murakami, Takahiro Arakawa, Takashi Kobayashi, Hiroshi Inui, Sakae Tanaka

**Affiliations:** ^1^ Department of Orthopaedic Surgery, Faculty of Medicine The University of Tokyo Bunkyo‐ku Tokyo Japan; ^2^ Graduate School of Health Sciences Morinomiya University of Medical Sciences Osaka Japan; ^3^ Department of Information Systems, Faculty of Engineering Saitama Institute of Technology Fukaya Saitama Japan; ^4^ Department of Orthopaedic Surgery, Saitama Medical Center Saitama Medical University Kawagoe City Saitama Japan

**Keywords:** in vivo kinematics, medial pivot total knee arthroplasty, valgus knees, varus knees

## Abstract

**Purpose:**

The efficacy of medial pivot total knee arthroplasty (MP TKA) in treating valgus knees that may cause medial instability is unknown. The purpose of this study was to investigate the in vivo kinematics of MP TKA for the valgus knees and compare them to those for the varus knees.

**Methods:**

The kinematics of 19 valgus knees and 19 varus knees operated in the MP TKA were investigated under fluoroscopy during squatting using a two‐ to three‐dimensional registration technique. Accordingly, the valgus and varus knees were evaluated and compared in terms of knee flexion angle, anteroposterior translation for the medial and lateral low contact points, axial rotation and valgus–varus angle of the femoral component relative to the tibial component, as well as kinematic pathways.

**Results:**

The knee flexion angle was found to be identical in both knees. There was no anterior translation on the medial side of the valgus knees, and no difference was detected between the two knees. On the lateral side, posterior translation was observed in both knees, with no difference between the two. Femoral external rotation was observed in both knees, and no difference was detected between the two. There was no valgus–varus change in the valgus knees, nor was there a difference between the two knees. The valgus knees demonstrated MP motion, whereas the varus knees demonstrated MP motion and bicondylar rollback.

**Conclusion:**

The medial side of the valgus knees treated with MP TKA showed comparable stable kinematics to the varus knees. The MP TKA is an effective procedure for valgus knees to stabilize the medial compartment.

**Level of Evidence:**

Level Ⅲ.

**Abbreviations**
2D: two‐dimensional
3D: three‐dimensional
AP: anteroposterior
HKA: hip‐knee‐ankle
KOOS: Knee Injury and Osteoarthritis Outcome Score
KSS: Knee Society Score
LCS: local coordinate system
MP: medial pivot
OA: osteoarthritis
PROM: patient‐reported outcome measures
TKA: total knee arthroplasty

## INTRODUCTION

Valgus knee deformity accounts for approximately 10% of patients requiring total knee arthroplasty (TKA) [19]. TKA for valgus knee deformity is occasionally difficult because the valgus knee has medial instability and lateral soft‐tissue tightness depending on the degree of deformity [12, 15]. To control the medial instability and achieve medial stability, a medial pivot (MP) TKA implant could be a viable option because of the high conformity (ball in socket) of the medial compartment. Previous research found that MP TKA is more stable in mid‐flexion than posterior stabilized TKA or cruciate retaining TKA [18]. Regarding clinical outcomes, MP TKA for patients with valgus knee osteoarthritis (OA) was as effective as MP TKA for patients with varus knee OA [10].

In terms of the kinematics of MP TKA for valgus deformity, intraoperative kinematics have been reported. Yamagami et al. found that intraoperative femoral rotational kinematics was comparable in valgus and varus knees during MP TKA [27]. However, the research on the in vivo kinematics of MP TKA for valgus knees is limited. Furthermore, it is unclear whether the MP TKA for valgus knees has stable in vivo kinematics, whereas the MP TKA for varus knees does [11]. The purpose of this study was to investigate the in vivo kinematics of MP TKA for the valgus knees and compare them to those for the varus knees. The hypothesis is that MP TKA for the valgus knees would exhibit stable in vivo kinematics similar to MP TKA for the varus knees.

## METHODS

This study was authorized by our institutional review board [number 10462‐(2)], and all patients gave their written informed consent. This study examined 19 valgus knees in 16 patients and 19 varus knees in 15 patients who underwent TKA at our institution between December 2018 and February 2023. The patients were specifically selected for this study according to the following criteria. The inclusion and exclusion criteria for valgus and varus knees are listed in Table 1. The selected knees were operated with the MP TKA implant (GMK Sphere; Medacta International), and all patients gave their consent for fluoroscopic evaluation. Valgus knees had a hip‐knee‐ankle (HKA) angle >180° and the alignment could be corrected with varus stress. Fixed valgus knees were excluded. The varus knees had a HKA angle <180°. Table 2 displays patient demographics. The HKA angle was determined using full‐length standing radiographic images. The knee extension and flexion angles were measured using a goniometer. The radiographic femoral and tibial component positions (*α*, *β*, *γ* and *δ* angles) were evaluated using the Knee Society's TKA Roentgenographic Evaluation [5]. The Knee Injury and Osteoarthritis Outcome Score (KOOS) and 2011 Knee Society Score (KSS) were evaluated pre‐operatively (Table 3) and at 1 year postoperatively (Table 4) as the patient‐reported outcome measures (PROMs). Additionally, the improvement of scores, defined as post‐operative score minus pre‐operative score, was evaluated (Table 5). The KOOS and 2011 KSS are valid and reliable outcome measures for TKA patients [17, 20, 22]. Higher scores indicate better knee condition.

**Table 1:** Inclusion and exclusion criteria for valgus and varus knees.

	Valgus knees	Varus knees
Inclusion	HKA angle >180°	HKA angle <180°
	Correctable with varus stress (not fixed valgus knees)	
	Kellgren–Lawrence OA Grade ≥3	
	Primary TKA with MP type	
	Post‐operative knee flexion angle ≥110°	
Exclusion	Post‐traumatic OA	
	Inflammatory arthritis (e.g., rheumatoid arthritis)	
	Revision TKA	
	Post‐operative knee flexion angle <110°	
	Primary TKA with other types (not MP type)	
	No consent for the fluoroscopic evaluation	

Abbreviations: HKA, hip‐knee‐ankle; MP, medial pivot; OA, osteoarthritis; TKA, total knee arthroplasty.

**Table 2:** Patient demographic characteristics.

	Valgus knees (*n* = 19)	Varus knees (*n* = 19)	*p* Values
Sex (female/male)	15/4	14/5	1.000
Age (years)	74.7 ± 7.2	77.2 ± 6.3	0.28
Body height (cm)	157.0 ± 7.6	152.9 ± 8.2	0.129
Body weight (kg)	62.9 ± 10.0	61.4 ± 9.6	0.642
Body mass index (kg/m^2^)	25.5 ± 3.7	26.2 ±± 3.4	0.552
Pre‐operative hip‐knee‐ankle angle (°)	190.9 ± 6.5	168.5 ± 5.4	**<0.001**
Pre‐operative maximum extension (°)	−6.4 ± 5.8	−8.7 ± 5.2	0.216
Pre‐operative maximum flexion (°)	122.4 ± 11.6	116.8 ± 9.1	0.120
Post‐operative hip‐knee‐ankle angle (°)	179.3 ± 2.0	179.2 ± 2.2	0.900
Post‐operative maximum extension (°)	−1.8 ± 2.2	−2.3 ± 2.8	0.522
Post‐operative maximum flexion (°)	123.7 ± 7.0	118.1 ± 6.5	**0.019**
*α* angle (°)	95.1 ± 1.1	95.5 ± 1.4	0.354
*β* angle (°)	89.0 ± 1.1	89.0 ± 1.5	0.878
*γ* angle (°)	2.7 ± 1.4	2.7 ± 1.3	0.937
*δ* angle (°)	87.3 ± 1.7	88.0 ± 0.9	0.118
Timing of fluoroscopic survey after surgery (months)	11.6 ± 6.2	12.2 ± 5.5	0.769

*Note*: Data are expressed as means ± standard deviations. The bold type indicates significance.

**Table 3:** Pre‐operative KOOS and 2011 KSS.

	Valgus knees (*n* = 19)	Varus knees (*n* = 19)	*p* Values
KOOS			
Pain	44.9 ± 22.1	51.2 ± 16.4	0.275
Symptoms	44.9 ± 22.0	55.4 ± 17.9	0.118
Function in daily living activities	52.2 ± 19.1	56.1 ± 12.6	0.456
Function in sports and recreation	14.7 ± 20.5	18.9 ± 20.0	0.584
Quality of life	26.6 ± 18.7	29.9 ± 18.8	0.673
2011 KSS			
Symptoms	9.1 ± 5.9	9.2 ± 4.6	0.925
Satisfaction	13.5 ± 9.6	13.3 ± 5.2	0.947
Expectation	13.7 ± 1.5	12.5 ± 1.8	**0.032**
Functional activities	39.0 ± 23.5	43.3 ± 14.0	0.515

*Note*: Data are presented as means ± standard deviations. The bold type indicates significance.

Abbreviations: KOOS, Knee Injury and Osteoarthritis Outcome Score; KSS, Knee Society Score.

**Table 4:** Post‐operative KOOS and 2011 KSS.

	Valgus knees (*n* = 19)	Varus knees (*n *= 19)	*p* Values
KOOS			
Pain	84.3 ± 16.1	87.8 ± 9.5	0.493
Symptoms	85.9 ± 13.0	85.4 ± 14.5	0.742
Function in daily living activities	80.8 ± 15.6	84.8 ± 7.5	0.376
Function in sports and recreation	47.4 ± 22.0	46.1 ± 25.2	0.773
Quality of life	62.5 ± 18.3	64.7 ± 22.3	0.837
2011 KSS			
Symptoms	21.5 ± 2.7	19.9 ± 3.9	0.204
Satisfaction	30.1 ± 8.0	27.9 ± 6.7	0.397
Expectation	12.0 ± 2.3	9.7 ± 2.5	**0.012**
Functional activities	70.0 ± 20.4	69.4 ± 16.8	0.927

*Note*: Data are presented as means ± standard deviations. The bold type indicates significance.

Abbreviations: KOOS, Knee Injury and Osteoarthritis Outcome Score; KSS, Knee Society Score.

**Table 5:** Improvement of KOOS and 2011 KSS.

	Valgus knees (*n* = 19)	Varus knees (*n* = 19)	*p* Values
Improvement of KOOS			
Pain	40.7 ± 24.1	36.5 ± 16.4	0.455
Symptoms	42.5 ± 24.1	30.1 ± 15.7	0.059
Function in daily living activities	29.7 ± 14.0	28.7 ± 10.6	0.747
Function in sports and recreation	31.8 ± 23.0	27.2 ± 17.5	0.459
Quality of life	38.6 ± 18.3	34.9 ± 19.2	0.541
Improvement of 2011 KSS			
Symptoms	12.3 ± 6.1	10.7 ± 5.0	0.441
Satisfaction	17.1 ± 8.6	14.7 ± 8.2	0.429
Expectation	−1.9 ± 2.1	−2.8 ± 3.5	0.393
Functional activities	31.6 ± 17.5	25.0 ± 12.3	0.239

*Note*: Improvement means post‐operative score minus pre‐operative score. Data are presented as means ± standard deviations.

Abbreviations: KOOS, Knee Injury and Osteoarthritis Outcome Score; KSS, Knee Society Score.

All operations were performed by the same surgical team under the same expert senior surgeon, using the GMK Sphere implant and the image‐free navigation system (Precision N, Stryker Orthopaedics). The GMK Sphere implant was the posterior cruciate ligament‐sacrificing type and a fixed‐bearing insert with a ‘ball in socket’ medially and flat lateral surface. The surgery was performed using a medial parapatellar approach, except for two cases where a lateral parapatellar approach was used due to severe deformity. The patella was not everted. The coronal alignment of the distal femur and proximal tibia was set to be perpendicular to the mechanical axis. The sagittal alignment of the distal femur was performed at four degrees of flexion to avoid anterior notching [16]. The sagittal alignment of the proximal tibia was performed at three degrees of flexion. The femoral axial rotational alignment was determined to be parallel to the surgical epicondylar axis, while the tibial axial rotational alignment was determined by referring to the Akagi line [1]. Additionally, to obtain extension–flexion balanced gaps, the soft‐tissue release of the iliotibial band and popliteus tendon was performed for valgus knees [21]. Following implantation and temporary joint capsule closure, patellar tracking was checked and lateral retinacular release was performed if necessary. Following the surgery, the post‐operative rehabilitation followed the same protocol in all cases.

**Figure 1:**
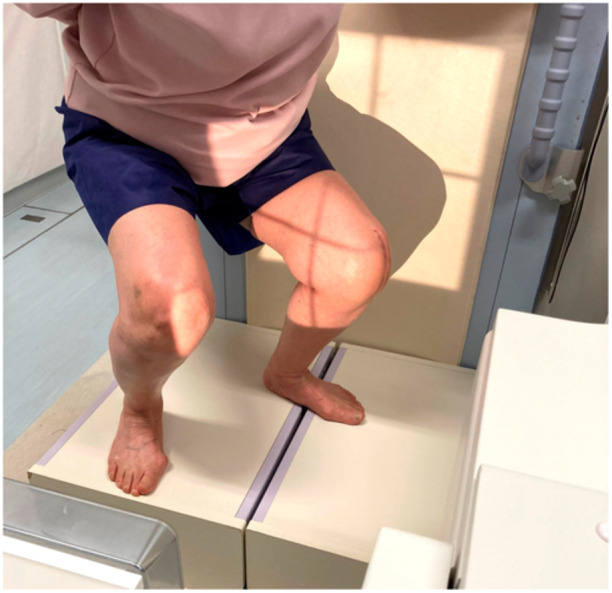
Under fluoroscopy, each patient squatted at their natural pace.

For the in vivo kinematic evaluation, each patient squatted under single‐view fluoroscopy in the sagittal plane [14, 23] (Figure 1). The squatting was done at a natural pace, from maximum extension to maximum flexion, according to a previously described method [23]. The sequential motion was recorded by digital radiographic images (1024 × 1024 × 12 bits/pixel, 7.5 Hz serial spot images saved in the digital imaging and communications in medicine file formats), with a 17‐in. flat panel detector system (ZEXIRA DREX‐ZX80; Toshiba). All images were processed with dynamic range compression to acquire edge‐enhanced images. A two‐dimensional to three‐dimensional (2D/3D) registration technique was employed to estimate the spatial position and orientation of the femoral and tibial components [28, 29, 30]. This technique employs a contour‐based registration algorithm for 2D single‐view fluoroscopic images and 3D computer‐aided design models. The accuracy of relative motion between the femoral and tibial components was estimated to be ≤0.4 mm for translations and ≤0.5° for rotations [29]. A local coordinate system (LCS) of the components was established using a previously described method [23, 29]. The femoral component's LCS originated at its gravity centre, while the tibial component's LCS originated at the centre of the tibial tray's surface. The following kinematic parameters were evaluated:

- Knee flexion angle between femoral and tibial components.
- Anteroposterior (AP) translation of the medial and lateral femorotibial low contact points.
- Axial rotational angle of the femoral component relative to the tibial component.
- Valgus–varus angle of the femoral component relative to the tibial component.
- Kinematic pathway.

**Figure 2:**
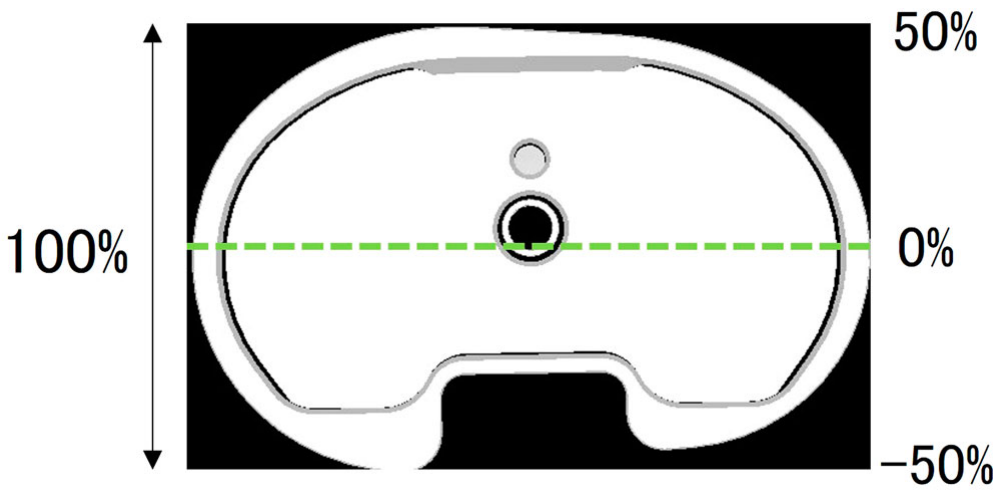
Anteroposterior (AP) length of the tibial tray. The AP translation was expressed as a percentage relative to the AP length of the tibial tray. The anterior location of the femoral component relative to the tibia was denoted as a positive value.

The knee flexion, femoral rotational and valgus–varus angles were calculated using the joint's conventional rotational method described by Grood and Suntay [9]. The AP translation was calculated as a percentage of the AP length of the tibial tray (Figure 2). A positive value of AP translation was defined as the femoral component being located anterior to the origin of the LCS of the tibial component. The femoral external rotation and valgus angle relative to the tibia were defined as positive. Kinematic data were presented as mean ± standard deviation.

### Statistical analysis

The statistical analysis was performed with SPSS (version 25, IBM Corporation). The patient gender distribution was determined using Fisher's exact test. An unpaired *t*‐test was used to compare the valgus and varus knees based on patient demographics, KOOS, 2011 KOOS scores and knee flexion angles. Repeated measures of analysis of variance and post hoc pairwise comparisons (Bonferroni test) were used to assess the differences in the AP translation, rotational and valgus angle between the valgus and varus knees. A *p* value of <0.05 was deemed statistically significant. Prior to this study, a power analysis was performed using G*Power (version 3.1.9.4, Heinrich Heine University) [6]. Fourteen knees are required for the alpha set, power and effect size of 0.05, 0.8 and 0.25, respectively (Supporting Information S1: Table a). Additionally, the minimum detectable difference in each kinematic variable was calculated (Supporting Information S1: Table b).

## RESULTS

### Radiographic evaluation and PROM

The pre‐operative HKA angle was 190.9 ± 6.5° for valgus knees and 168.5 ± 5.4° for varus knees, which differed significantly (Table 2).

The pre‐operative and post‐operative expectation score of the 2011 KSS was significantly higher in valgus knees than in varus knees (Tables 3 and 4). The improvement of KOOS symptoms tended to be higher in valgus knees than in varus knees (Table 5).

### Knee flexion angle

The maximum extension angles for valgus and varus knees were 5.0 ± 7.1° and 4.4 ± 7.1°, respectively. The maximum flexion angles for valgus and varus knees were 113.1 ± 12.5° and 108.3 ± 10.7°, respectively. There was no significant difference between the valgus and varus knees in terms of the maximum extension and flexion angles.

### AP translation

**Figure 3:**
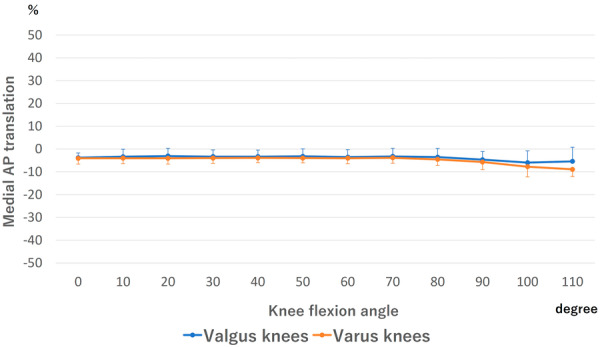
Anteroposterior (AP) translation of the medial side of the femoral component. There was no significant difference at all flexion angles between valgus and varus knees.

The medial AP translation of the femoral component is shown in Figure 3. No AP motion was observed from 0° to 110° in the valgus knees. In varus knees, AP motion was not observed from 0° to 70° and posterior translation was observed beyond 70°. No difference was detected at all flexion angles between valgus and varus knees.

**Figure 4:**
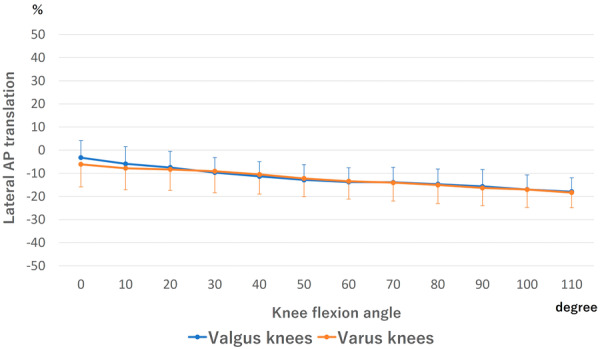
Anteroposterior (AP) translation of the lateral side of the femoral component. There was no significant difference at all flexion angles between valgus and varus knees.

The lateral AP translation of the femoral component is shown in Figure 4. The posterior translation was observed from 0° to 110° in both valgus and varus knees. No difference was detected at all flexion angles between valgus and varus knees.

### Rotational angle

**Figure 5:**
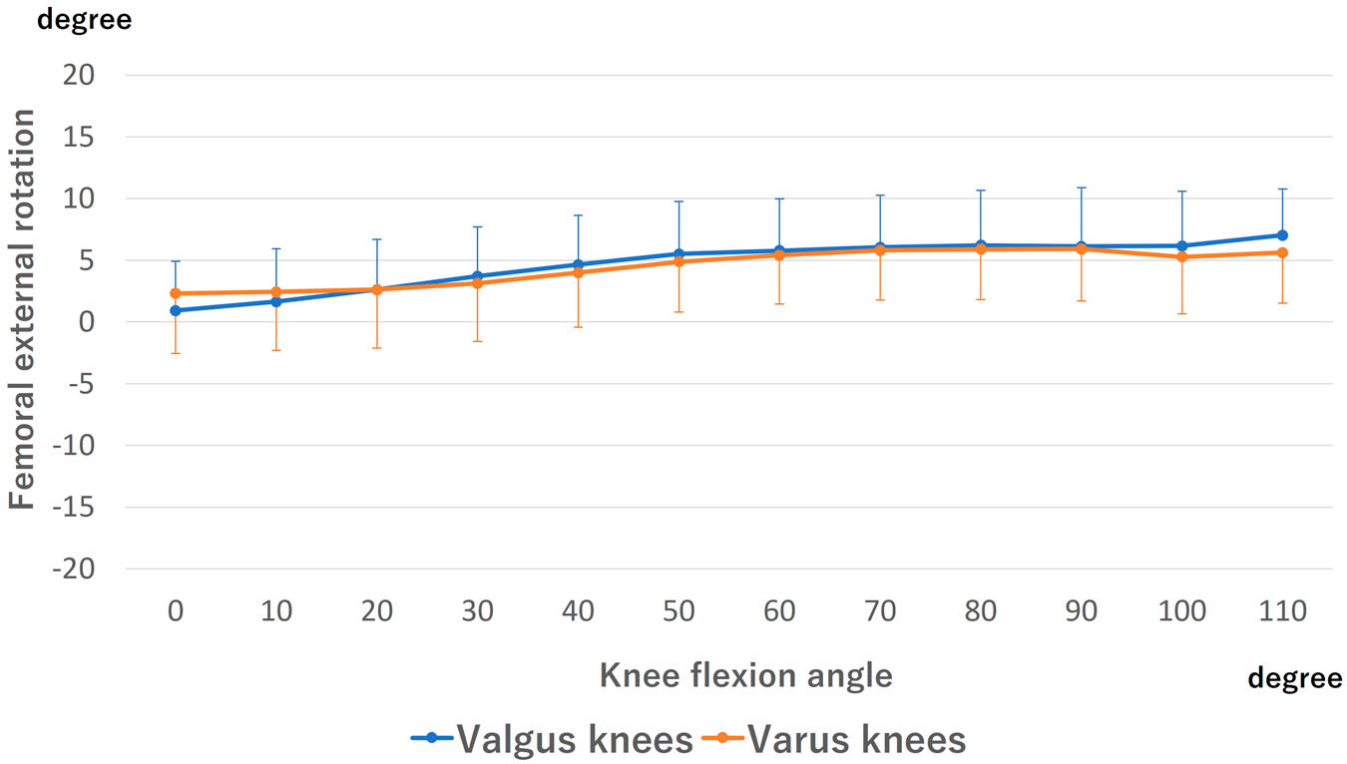
Rotation of the femoral component. The external rotation of the femur relative to the tibia was marked as positive. There was no significant difference at all flexion angles between valgus and varus knees.

Femoral external rotation is shown in Figure 5. The gradual external rotation was observed from 0° to 110° in both the valgus and varus knees. No difference was detected at all flexion angles between valgus and varus knees.

### Valgus–varus angle

**Figure 6:**
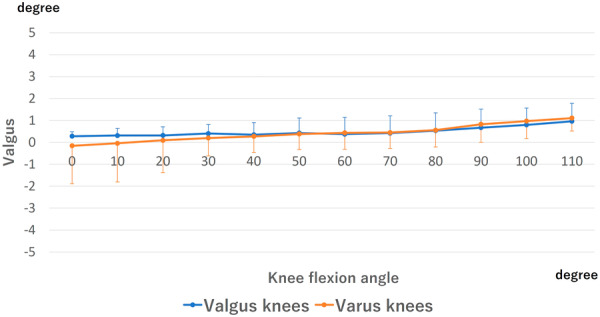
Valgus–varus angle of the femoral component. The valgus angle of the femur relative to the tibia was marked as positive. There was no significant difference at all flexion angles between valgus and varus knees.

Valgus–varus angle is shown in Figure 6. There was no valgus–varus change from 0° to 110° in the valgus knees. The varus knees showed no valgus–varus change from 0° to 30° followed by a 0.9 ± 0.7° valgus change from 30° to 110°. No difference was detected at all flexion angles between valgus and varus knees.

### Kinematic pathway

**Figure 7:**
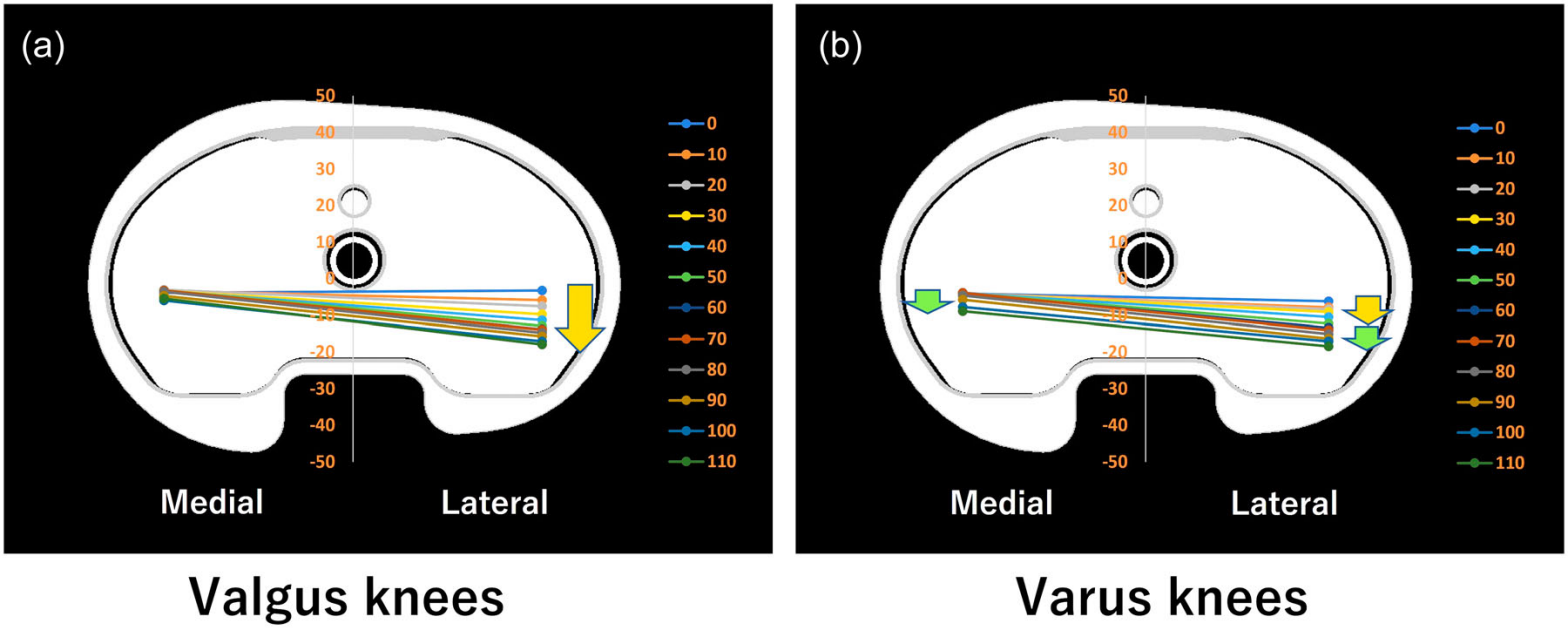
Kinematic pathway for valgus and varus knees. (a) Valgus knees exhibit medial pivot motion. (b) Varus knees exhibit medial pivot motion and bicondylar rollback.

Valgus knees demonstrated MP motion (Figure 7a). In the varus knees, MP motion was observed from 0° to 70° followed by bicondylar rollback beyond 70° (Figure 7b).

## DISCUSSION

The most important finding of this study was that the medial AP translation of the valgus knees was stable and comparable to that of the varus knees. To our knowledge, this is the first study to compare the in vivo kinematics of MP TKA of the valgus and varus knees. The finding of medial AP translation in this study differed from previous in vivo kinematic studies [13, 24]. Kitagawa et al. studied the postoperative in vivo kinematics of severe valgus deformity using an MP TKA (ADVANCE, Wright Medical Technology) and found a small amount of anterior translation of the medial side during weight‐bearing deep knee bend [13]. Furthermore, Suzuki et al. investigated the in vivo kinematics of valgus knees operated by posterior stabilized TKA, observing anterior translation of the medial side from full extension to 60° of flexion during squatting [24]. Paradoxical anterior movement is an anterior translation of the femur in the mid‐flexion range [3, 4]. The absence of the anterior cruciate ligament can cause paradoxical motion [8, 26]. Van Onsem et al. found that TKA patients in the low PROM group showed anterior translation on the medial side due to mid‐flexion instability [25]. In our study, no anterior translation was observed across all flexion angles. This suggests that the anterior stability of the medial side was achieved by the high conformity of the MP implant, even in the valgus knees as well as the varus knees.

Regarding the lateral AP translation and femoral rotation, this study's finding of valgus knees was consistent with previous research [13, 24]. This study found a posterior translation of the lateral side and femoral external rotation of approximately 5° from extension to flexion, which was consistent with previous findings [13, 24]. This physiological lateral posterior translation and femoral external rotation could have resulted from proper extension–flexion gap balancing and soft‐tissue release, even in the valgus knees.

In the valgus–varus angle, no difference was detected between the valgus and varus knees. The valgus knees achieved post‐operative neutral alignment despite having a pre‐operative HKA angle of 190.9 ± 6.5° (Table 2). In addition to proper surgical technique, the high conformity of the MP TKA implant may have helped achieve the desired neutral alignment.

Regarding the kinematic pathway, MP motion was observed not only in the varus knees but also in the valgus knees. Kitagawa et al. described the pre‐operative in vivo kinematics of valgus knees, finding that the medial posterior translation of the femur was greater than the lateral posterior translation from extension to flexion [13]. The post‐operative MP motion in the valgus knees in this study may have been induced by the MP implant designed for physiological MP motion [7], although the pre‐operative kinematics was not assessed in this study. Alesi et al. found that there was a correlation between the presence of MP motion and higher post‐operative clinical outcomes [2]. In this study, the post‐operative maximum flexion angle measured using a goniometer was significantly greater in valgus knees than in varus knees, although the pre‐operative maximum flexion angle did not differ significantly between valgus and varus knees. In terms of clinical score, the post‐operative expectation of the 2011 KSS was higher in valgus knees than in varus knees (Table 4). Furthermore, the improvement of KOOS symptoms tended to be greater in the valgus knees than in the varus knees (Table 5). Furthermore, the absolute scores of improvements were higher in the valgus knees on all KOOS and 2011 KSS subscales. Therefore, the acquisition of MP motion in the valgus knees may be associated with a higher post‐operative score in this study.

The study's strength was that it compared the valgus and varus knees using the same MP implant and investigated the in vivo kinematics of both knees under weight‐bearing conditions. To date, no study has been found that compares in vivo kinematics under weight‐bearing conditions between valgus and varus knees using the same MP implant. Yamagami et al. investigated the intra‐operative kinematics of MP TKA between the valgus and varus knees, and the femoral rotational kinematics were similar [27]. However, the study was conducted intra‐operatively in a non‐weight‐bearing condition. To assess weight‐bearing flexion activity as performed in daily life, an in vivo kinematic study under weight‐bearing conditions was required.

The limitations of this study should be noted. First, fixed valgus knees were excluded from this study. Therefore, the study's findings were limited to correctable valgus knees, and the kinematics of fixed valgus knees operated with this MP implant are unknown. Second, pre‐operative kinematics was not assessed in this study. If the pre‐operative kinematics of the same patients were evaluated, the difference between the pre‐operative and post‐operative kinematics could be clarified. Third, only the squatting motion was assessed in this study. If other motions performed in daily activities were also evaluated, the findings of this study could have been slightly different. For example, patients often report instability during stair ascent/descent. We are planning to evaluate the stair activity in the valgus and varus knees in future works.

## CONCLUSION

The medial side of the valgus knees treated with MP TKA showed comparable stable kinematics to the varus knees. MP TKA is an effective procedure for valgus knees to stabilize the medial compartment.

## AUTHOR CONTRIBUTIONS

Tomofumi Kage, Tetsuya Tomita, Takaharu Yamazaki, Shuji Taketomi, Ryota Yamagami, Kohei Kawaguchi, Ryo Murakami, Takahiro Arakawa, Takashi Kobayashi and Hiroshi Inui contributed study conception, design, data acquisition, analysis and interpretation. Tomofumi Kage and Kenichi Kono contributed to article drafting or critical revision. Kenichi Kono and Sakae Tanaka contributed final approval. All authors have read and approved the final manuscript.

## CONFLICT OF INTEREST STATEMENT

The authors have received grants from Medacta International and Stryker outside this study. No author has a conflict of interest regarding the topics discussed in this study.

## ETHICS STATEMENT

This study was approved by the institutional review board of the University of Tokyo　(IRB No: 10462‐(2)). Informed consent was obtained from all individual participants included in the study.

## Supporting information

Suppoting information.

## Data Availability

The data sets used and/or analyzed during the current study are available from the corresponding author upon reasonable request.
