# Supplementary material for: Medial pivot total knee arthroplasty for valgus knees provides equivalent medial stability compared to that for varus knees: In vivo kinematic study
Source: J Exp Orthop. 2024 Dec 18;11(4):e70013. doi: 10.1002/jeo2.70013 (PMC11655669; doi:10.1002/jeo2.70013)
Supplement: Supplementary file 1 — Suppoting information. [file JEO2-11-e70013-s001.docx]

**Supplementary Material**

**Table a.** Power analysis

|  |  |
| --- | --- |
| Effect size f | 0.25 |
| Α error probability | 0.05 |
| Power (1–β error probabity) | 0.80 |
| Number of groups | 2 |
| Number of measurements | 12 |
| Correlation among repeated measures | 0.5 |
| Nonsphericity correction coefficient | 1 |
|  |  |
| Total sample size | 14 |

**Table b.** Minimum detectable difference (MDD)

| Variable | ICC | SEM | **MDD** |
| --- | --- | --- | --- |
| Anteroposterior translation (mm) | 0.87 | 0.13 | 0.35 |
| Rotational angle (°) | 0.90 | 0.12 | 0.33 |
| Valgus-varus angle (°) | 0.95 | 0.02 | 0.06 |

ICC, intra-class coefficient; SEM, standard error of the mean; MDD, minimum detectable difference; SD, standard deviation.

SEM and MDD were calculated in the following formula.

SEM = SD × $\sqrt{(1-\mathrm{ICC})}$

MDD = SEM × 1.96 × $\sqrt{2}$

**Table 4.** Post-operative KOOS and 2011 KSS scores

|  | Valgus knees (n = 19) | Varus knees (n = 19) | *P*-values |
| --- | --- | --- | --- |
| KOOS |  |  |  |
| Pain | 84.3 ± 16.1 | 87.8 ± 9.5 | 0.493 |
| Symptoms | 85.9 ± 13.0 | 85.4 ± 14.5 | 0.742 |
| Function in daily living activities | 80.8 ± 15.6 | 84.8 ± 7.5 | 0.376 |
| Function in sports and recreation | 47.4 ± 22.0 | 46.1 ± 25.2 | 0.773 |
| Quality of life | 62.5 ± 18.3 | 64.7 ± 22.3 | 0.837 |
| 2011 KSS |  |  |  |
| Symptoms | 21.5 ± 2.7 | 19.9 ± 3.9 | 0.204 |
| Satisfaction | 30.1 ± 8.0 | 27.9 ± 6.7 | 0.397 |
| Expectation | 12.0 ± 2.3 | 9.7 ± 2.5 | **0.012** |
| Functional activities | 70.0 ± 20.4 | 69.4 ± 16.8 | 0.927 |

KOOS, Knee injury and Osteoarthritis Outcome Score; 2011 KSS, 2011 Knee Society Score

Data is presented as means ± standard deviations. The bold type indicates significance.

**Table 5.** Improvement of KOOS and 2011 KSS scores

|  | Valgus knees (n = 19) | Varus knees (n = 19) | *P*-values |
| --- | --- | --- | --- |
| Improvement of KOOS |  |  |  |
| Pain | 40.7 ± 24.1 | 36.5 ± 16.4 | 0.455 |
| Symptoms | 42.5 ± 24.1 | 30.1 ± 15.7 | 0.059 |
| Function in daily living activities | 29.7 ± 14.0 | 28.7 ± 10.6 | 0.747 |
| Function in sports and recreation | 31.8 ± 23.0 | 27.2 ± 17.5 | 0.459 |
| Quality of life | 38.6 ± 18.3 | 34.9 ± 19.2 | 0.541 |
| Improvement of 2011 KSS |  |  |  |
| Symptoms | 12.3 ± 6.1 | 10.7 ± 5.0 | 0.441 |
| Satisfaction | 17.1 ± 8.6 | 14.7 ± 8.2 | 0.429 |
| Expectation | –1.9 ± 2.1 | –2.8 ± 3.5 | 0.393 |
| Functional activities | 31.6 ± 17.5 | 25.0 ± 12.3 | 0.239 |

Improvement means post-operative score minus pre-operative score.

KOOS, Knee injury and Osteoarthritis Outcome Score; 2011 KSS, 2011 Knee Society Score

Data is presented as means ± standard deviations.
